# General knowledge and practice of household dog owners regarding gastrointestinal parasites in Cebu, Philippines

**DOI:** 10.14202/vetworld.2023.1438-1443

**Published:** 2023-07-09

**Authors:** Marysia Frances M. Urgel, Rochelle Haidee D. Ybañez, Adrian P. Ybañez, Elmie S. Delan

**Affiliations:** 1 Department of Biology, College of Science, University of the Philippines Cebu, Gorordo Avenue, Lahug, Cebu City, 6000 Cebu, Philippines; 2 National Research Center for Protozoan Diseases, Obihiro University of Agriculture and Veterinary Medicine, Obihiro City, Hokkaido, Japan; 3 College of Veterinary Medicine, Cebu Technological University-Barili Campus, Cagay, 6036 Barili, Philippines; 4 Institute for Molecular Genetics, Parasitology and Vector-Borne Diseases, Cebu Technological University-Main Campus, Cebu City, 6000 Cebu, Philippines

**Keywords:** dogs, intestinal parasites, soil, vaccination

## Abstract

**Background and Aim:**

Canine gastrointestinal tract (GIT) parasites are of public health and veterinary concern and are harmful to both humans and animals. The risk of transmitting GIT parasites can be minimized depending on dog owners’ knowledge and management practices. Therefore, this study aimed to assess dog owners’ general knowledge and practices regarding GIT parasites.

**Materials and Methods:**

A structured questionnaire containing 10-point Likert statements was administered to 130 respondents in Cebu, Philippines, to obtain information about their profile (age, sex, civil status, and educational attainment) and data regarding their home environment, number of dogs and other pets owned, and their knowledge and practices regarding canine GIT parasites. These respondents had previously provided canine stool samples for fecalysis.

**Results:**

Most respondents were female (65.4%), 18–24 years old (55.4%), single (71.5%), and educated to college level (49.2%). Housing styles were mostly gated (76.9%), and >50% had a garden. The majority owned 1–3 dogs (56.9%) and did not own any other pets (73.8%). All knew about canine GIT parasites. There was a significant association between GIT parasite positivity in dogs and the owner’s sex (p = 0.005). Gastrointestinal parasite positivity was also associated with the owner’s knowledge about the transmission of parasites from the mother’s milk to her puppies (p = 0.001), canine intestinal parasites potentially causing weight loss, diarrhea, and vomiting (p = 0.001), and dogs potentially becoming infected with parasites from licking or sniffing the soil or grass where other animals have been (p = 0.005). Moreover, there was a significant association between GIT parasite positivity and the owner’s practice of daily cleaning of the areas where the dog defecated (p = 0.001), deworming every 6–12 months (p = 0.001), and unfailingly following the vaccination and deworming schedule (p = 0.006). Finally, the summative knowledge and practice level of the owners were significantly associated with GIT parasite positivity (p = 0.001).

**Conclusion:**

This study highlights the need for continuous education of dog owners to maintain good knowledge and practices to prevent GIT parasite infection. Frequent deworming (once a month) of adult dogs is recommended.

## Introduction

Pet dogs live closely with their human owners, contributing to each other’s overall well-being [1]. They provide companionship and security while owners provide the care the dog needs [2, 3]. However, dogs (whether sheltered or stray) are important reservoir hosts to various ecto-and endoparasites, particularly gastrointestinal tract (GIT) protozoa and helminths [4, 5].

Canine GIT parasites are of veterinary and public health importance and cause morbidity in dogs and humans [6]. Infection can be subclinical but can manifest in dogs with health concerns [7]. Depending on the parasite density and type, GIT parasites cause different clinical symptoms, some of which could be fatal, particularly in severe cases when left untreated [8]. Despite the availability of medications for treatment, parasite elimination is impossible due to the different stages in its life cycle [9]. Moreover, infestation symptoms may not manifest until the infection is sufficiently severe [10]. In Cebu, Philippines, detected canine GIT parasites include *Ancylostoma*, *Trichuris*, and *Toxocara* spp. [11].

Pet owners may be unaware that their dogs may carry zoonotic parasites and be unfamiliar with their modes of transmission [3]. Without knowledge of the risks of zoonotic disease in their pets, owners are unlikely to be aware of the need for preventive measures to minimize risks [12]. This lack of awareness of the transmission of zoonotic diseases poses a serious public health risk [13]. Animal health and the environment are connected to public health [1].

To the best of the author’s knowledge, no recent studies in Cebu, Philippines, have assessed the knowledge and practices of household dog owners in relation to the presence of GIT parasites in their respective dogs. Thus, this study was conducted.

## Materials and Methods

### Ethical approval and Informed consent

The study was conducted according to the principles of the Helsinki Declaration developed by the World Medical Association. The protocol was approved by the University of the Visayas – Institutional Review Board. Written informed consent was obtained from the respondents.

### Study period and location

The study was conducted from January 2017 to May 2017 in Cebu, Philippines.

### Respondents

The study participants included 130 dog owners from different households aged 18–64 years and above from Cebu, Philippines. Only respondents who had previously collected a stool sample from their dogs for fecalysis in February to May 2017 were included in the study. Hence, the census method of data collection was employed.

### Questionnaire

A structured questionnaire was administered to gather information about the owners’ profiles, such as age, sex, civil status, and educational attainment, and data about their home environment and the number of dogs and other pets owned. The questionnaire also assessed the owner’s general knowledge of GIT parasites as follows: (1) potential transmission of parasites to dogs by other animals, (2) transmission of parasites to puppies through their mother’s milk, (3) transmission of parasites from dogs and humans through the fecal-oral route, (4) intestinal parasites in dogs causing weight loss, diarrhea, and vomiting, and (5) dogs obtaining parasites from licking or sniffling the soil or grass where other animals have been. The dog owners were also asked about the following practices related to GIT parasites: (1) feeding raw meat, (2) daily cleaning of areas where the dog had defecated, (3) deworming every 6–12 months, (4) following the vaccination and deworming appointments for their dogs as set by the veterinarian, and (5) maintaining a clean environment where the dogs usually stay. Before administration, the questionnaires were subjected to face validation by three identified experts and pretested on 25 respondents. The Cronbach alpha value was computed (0.64) and deemed acceptable.

The 10-point Likert scale answers were scored. The summative scores obtained by the owners were categorized and given a score of 5 (very high) if they scored 41–50 points, 4 (high) if they scored 32–40 points, 3 (moderate) if they scored 23–31 points, 2 (low) if they obtained scored 15–22 points, and 1 (very low) if scored below 15. On the other hand, the number of infected dogs and the specific parasite prevalence rates were estimated.

### Statistical analysis

The data were encoded in Microsoft Excel spreadsheets and analyzed using descriptive statistics. Chi-square test was used to determine the statistical significance between the owner’s profile, knowledge, and practices and the rate of GIT parasite positivity of their respective dogs. Results were considered significant when p ≤ 0.05.

## Results and Discussion

### Respondents’ profile

More than half of the respondents were 18–24 years old (55.4%), with an average age of 29.9 years. Most were female (65.4%), predominantly single (71.5%), and educated to college level (49.2%). Concerning housing styles, 76.9% were gated, and >50% had a lawn or garden. Finally, over half of the households owned one to three dogs (56.9%), and the majority did not own any other animals (73.8%) (Table-1). The owner’s age, gender, marital status, educational attainment, income [14, 15], and house ownership [16] are already established factors influencing dog ownership.

**Table-1:** Profile of dog owners interviewed for their knowledge and practices on gastrointestinal tract parasites in Cebu, Philippines (n=130).

Parameter	Frequency	Percentage
Age (years)		
18–24	72	55.4
25–44	27	20.8
45–64	27	20.8
Above 64	4	3.1
Mean=29.9		
SD=14.4		
Sex		
Male	45	34.6
Female	85	65.4
Civil status		
Married	37	28.5
Single	93	71.5
Highest educational attainment		
Elementary graduate	4	3.1
High school level	1	0.8
High school graduate	1	0.8
College level	64	49.2
College graduate	60	46.2
Housing characteristics		
Has a garden/lawn	4	3.1
All concrete ground	15	11.5
Gated house	44	33.8
Open house	9	6.9
Garden and gated house	46	35.4
Concrete and gated house	10	7.7
Garden and open house	2	1.5
Number of dogs owned		
1–3	74	56.9
4–6	43	33.1
7–9	13	10
Other pets owned		
No other animals	96	73.8
Cats	12	9.2
Birds	3	2.3
Others	16	12.3
All of the above	3	2.3
Number of other pets owned		
No other animals	96	73.8
1–3	13	10
4–6	4	3.1
7–9	4	3.1
10–12	4	3.1
13–15	9	6.9

*SD=Standard deviation

### General knowledge about canine GIT parasites

Regarding the knowledge of dog owners about GIT parasites, most were either undecided or did not agree that other animals can transmit parasites to dogs. Most were undecided or agreed that puppies can become infected with parasites from their mother’s milk. The majority were aware that GIT parasites can be transmitted to humans through the fecal-oral route; that they can cause weight loss, diarrhea, and vomiting; and that dogs can become infected with parasites from licking or sniffling the soil or grass where other animals have been. With the different statements, there were undecided respondents (10.8–43.8) concerning basic relevant facts about GIT parasites. Regarding the practices, almost all respondents did not feed their dogs raw meat. Most cleaned the areas where their dogs defecated. Most dewormed their dogs every 6–12 months and unfailingly followed the vaccinations and deworming appointments the veterinarian had set for their dogs. The importance of maintaining a clean environment where the dogs usually stay was almost recognized (Table-2).

**Table-2:** Dog owner’s general knowledge and practice on canine parasites in Cebu, Philippines (n = 130).

Statements	Description	Frequency	Percentage
General Knowledge			
S3. Other animals (cats, birds, etc.) CANNOT transmit parasites to my dogs	Strongly disagree	20	15.4
	Disagree	47	36.2
	Undecided	38	29.2
	Agree	14	10.8
	Strongly agree	11	8.5
S4. Puppies can get parasites from the milk of their mother	Strongly disagree	2	1.5
	Disagree	7	5.4
	Undecided	57	43.8
	Agree	58	44.6
	Strongly agree	6	4.6
S6. Parasites from dogs CANNOT be transmitted to humans through the oral-fecal route	Strongly disagree	21	16.2
	Disagree	55	42.3
	Undecided	35	26.9
	Agree	10	7.7
	Strongly agree	9	6.9
S7. Intestinal parasites in dogs can cause weight loss, diarrhea, and vomiting	Strongly disagree	--	--
	Disagree	--	--
	Undecided	14	10.8
	Agree	52	40
	Strongly agree	64	49.2
S10. Dogs can get parasites from licking or sniffling the soil or grass where other animals have been	Strongly disagree	2	1.5
	Disagree	1	0.8
	Undecided	26	20
	Agree	69	53.1
	Strongly agree	32	24.6
Practices			
S1. I do NOT feed my dog raw meat	Strongly disagree	--	--
	Disagree	2	1.5
	Undecided	6	4.6
	Agree	42	32.3
	Strongly agree	80	61.5
S2. I daily clean the areas where my dog has defecated	Strongly disagree	1	0.8
	Disagree	4	3.1
	Undecided	4	3.1
	Agree	45	34.6
	Strongly agree	76	58.5
S5. My dog is dewormed every 6–12 months	Strongly disagree	11	8.5
	Disagree	32	24.6
	Undecided	20	15.4
	Agree	39	30
	Strongly agree	28	21.5
S8. I religiously follow the vaccination and deworming appointments the vet has set for my dog	Strongly disagree	5	3.8
	Disagree	26	20
	Undecided	34	26.2
	Agree	37	28.5
	Strongly agree	28	21.5
S9. It is NOT important to maintain a clean environment where my dogs usually stay	Strongly disagree	82	63.1
	Disagree	44	33.8
	Undecided	--	--
	Agree	1	0.8
	Strongly agree	3	2.3

**Level of Knowledge and Practice (Summative Score-Category)**	**Frequency**	**Percentage**	**Number of respondents with dogs positive for GIT parasites**

Moderate (3.0)	5	3.8	2
High (4.0)	74	56.9	47
Very high (5.0)	51	39.2	10

GIT = Gastrointestinal tract

Overall, the knowledge and practices of most owners scored high (56.9%) and very high (39.3%). The dog owners appeared well informed, which may be attributed to better access to veterinary services and better living standards of owners [17]. Although the owners had good knowledge about canine GIT parasites, the number of dogs infected with parasites was still high (Table-2). The high prevalence may be due to environmental conditions. Defecation of dogs positive for GIT parasites in the garden may contaminate the soil, resulting in reinfection [18, 19]. This may even occur after deworming because GIT parasite eggs can remain stable in the environment for some time. On the other hand, management practices and deworming routines also influence GIT parasitism in dogs [13]. Another possibility is that deworming every 6–12 months may be insufficient to prevent GIT parasitism in dogs. The Tropical Council for Companion Animal Parasites recommends monthly deworming in adult dogs in tropical countries such as the Philippines [20].

There was a significant association between dogs positive for GIT parasites and their owner’s sex (p = 0.005). The dog owner’s sex may be associated with the severity of GIT parasitism, with those owned by females having higher tendencies to harbor GIT parasites [21]. However, our finding may be coincidental because most of the dog owners in this study were female.

Several knowledge factors were associated with GIT parasite positivity in dogs, including the owner’s knowledge about puppies becoming infected with parasites from their mother’s milk (p = 0.001). Transplacental and transmammary parasitic transmission from the mother to her puppies are possible [22, 23]. Thus, deworming of nursing mothers may help to reduce the risk of GIT parasite transmission.

Knowledge about GIT parasites potentially causing weight loss, diarrhea, and vomiting (p = 0.001) was associated with the GIT parasite positivity in the dogs. These are commonly observed clinical signs in dogs with parasites [8, 24]. Owners have a tendency to deworm their dogs if they observe the aforementioned signs.

Knowledge about dogs potentially becoming infected with parasites from licking or sniffing the soil or grass where other animals have been (p = 0.005) and the owner’s practice of daily cleaning of the areas where the dog has defecated (p = 0.001) was significantly associated with GIT parasite positivity. Soil contact is an identified risk factor [1]. This may be due to GIT parasite-positive dogs with unrestricted movement defecating in the soil, contaminating it with parasite eggs and oocysts [25]. This could favor zoonotic transmission and the (re-)infection of other dogs [19, 26]. On the other hand, owners who regularly clean the areas, particularly those contaminated with feces, can be critical in preventing the spread of GIT parasites [27]. The practice of proper disposal and follow-up cleaning of animal feces from the ground (parks, etc.) are important factors in minimizing environmental contamination and the risk of zoonotic transmission [28].

The practice of deworming the dogs every 6–12 months was significantly associated with GIT parasite positivity (p = 0.001). Deworming is critical in preventing GIT parasitism [13], and this practice has been recommended every 3–6 months [29]. Dogs that are dewormed at intervals of more than 6 months have a higher probability of GIT parasite infection [30]. However, the usual practice in the Philippines is the annual deworming of dogs when they receive their annual vaccinations (personal communication).

Owners who unfailingly follow vaccination and deworming appointments for their dogs with the veterinarian were also significantly associated with GIT parasite positivity (p = 0.006). Frequent veterinary visits can motivate dog owners to comply with recommended GIT prevention and control measures, including deworming and proper feces disposal [21]. The routine use of control products for intestinal parasites and fecal examinations allows prompt treatment of the affected dogs [7]. A lack of proper veterinary care for dogs increases the public health risk of GIT parasites [13].

Overall, the summative knowledge and practices of the dog owners were significantly associated with the presence of GIT parasites (p = 0.001) (Table-3). In other studies, pet owners’ knowledge of zoonoses and their practices were related to the zoonotic disease risks posed by their pets [12]. In addition, GIT parasite infection in dogs can be influenced by the owner’s management and deworming practices [13].

**Table-3:** Significant statistical analyses result between the owner’s profile, knowledge, and practice on canine parasites and the presence of GIT parasites in their dogs in Cebu, Philippines.

Parameter	Degrees of freedom (df)	χ^2^	p-value
Dog owner’s sex	1	7.872	0.005
Practice on daily cleaning of the areas where dog defecated	4	11.567	0.001
Knowledge about puppies getting parasites from the milk of their mother	4	11.768	0.001
Practice on deworming every 6–12 months	4	14.734	0.001
Knowledge about intestinal parasites in dogs can cause weight loss, diarrhea, and vomiting	4	11.156	0.001
Practice on religiously following the vaccination and deworming appointments the veterinarian set for the dog	4	7.47	0.006
Knowledge about dogs getting parasites from licking or sniffling the soil or grass where other animals have been	4	7.949	0.005
Overall knowledge and practice level	4	19.046	0.001

GIT=Gastrointestinal tract

## Conclusion

Maintaining good knowledge and practices for the prevention and control of GIT parasites in dogs are essential. Dog owners should be continuously educated about proper dog management, particularly for GIT parasites.

## Recommendations

Dog owners should continue to sustain good practices and remain up to date with the latest knowledge and products for GIT parasite prevention and control. In addition, frequent deworming (once a month) of adult dogs is recommended.

## Authors’ Contributions

APY, MFMU, and RHDY: Equal authors and conceptualized the study, conducted the research and finalized the manuscript. ESD: Data analysis and drafted the manuscript. All the authors have read, reviewed, and approved the final manuscript.

## References

[1] Overgaauw P.A, Vinke C.M, van Hagen M.A, Lipman L.J (2020). A one health perspective on the human-companion animal relationship with emphasis on zoonotic aspects. Int. J. Environ. Res. Publ. Health.

[2] McNicholas J, Gilbey A, Rennie A, Ahmedzai S, Dono J.A, Ormerod E (2005). Pet ownership and human health:A brief review of evidence and issues. BMJ.

[3] Gray P.B, Young S.Mssssw (2011). Human-pet dynamics in cross-cultural perspective. Anthrozoös.

[4] Paul M, King L, Carlin E.P (2010). Zoonoses of people and their pets:A US perspective on significant pet-associated parasitic diseases. Trends Parasitol.

[5] Robertson I.D, Irwin P.J, Lymbery A.J, Thompson R.C.A (2000). The role of companion animals in the emergence of parasitic zoonoses. Int. J. Parasitol.

[6] Otranto D, Dantas-Torres F, Mihalca A.D, Traub R.J, Lappin M, Baneth G (2017). Zoonotic parasites of sheltered and stray dogs in the era of the global economic and political crisis. Trends Parasitol.

[7] Rojekittikhun W, Chaisiri K, Mahittikorn A, Pubampen S, Sa-Nguankiat S, Kusolsuk T, Mori H (2014). Gastrointestinal parasites of dogs and cats in a refuge in Nakhon Nayok, Thailand. Southeast Asian J. Trop. Med. Public Health.

[8] Traub R.J, Pednekar R.P, Cuttell L, Porter R.B, Rani P.A.A.M, Gatne M.L (2014). The prevalence and distribution of gastrointestinal parasites of stray and refuge dogs in four locations in India. Vet. Parasitol.

[9] Stafford K, Kollasch T.M, Duncan K.T, Horr S, Goddu T, Heinz-Loomer C, Little S.E (2020). Detection of gastrointestinal parasitism at recreational canine sites in the USA:The DOGPARCS study. Parasit. Vectors.

[10] Lorenzini G, Tasca T, De Carli G.A (2007). Prevalence of intestinal parasites in dogs and cats under veterinary care in Porto Alegre, Rio Grande do Sul, Brazil. Braz. J. Vet. Res. Anim. Sci.

[11] Zewdu E, Semahegn Y, Mekibib B (2010). Prevalence of helminth parasites of dogs and owners awareness about zoonotic parasites in Ambo town, central Ethiopia. Ethiop. Vet. J.

[12] Paulos D, Addis M, Fromsa A, Mekibib B (2012). Studies on the prevalence of gastrointestinal helminthes among dogs and owners perception about zoonotic dog parasites in Hawassa Town, Ethiopia. J Public Health Epidemiol.

[13] Urgel M.F.M, Ybañez R.H.D, Ybañez A.P (2019). The detection of gastrointestinal parasites in owned and shelter dogs in Cebu, Philippines. Vet. World.

[14] Westgarth C, Pinchbeck G.L, Bradshaw J.W, Dawson S, Gaskell R.M, Christley R.M (2007). Factors associated with dog ownership and contact with dogs in a UK community. BMC Vet. Res.

[15] Bir C, Widmar N.J.O, Croney C.C (2017). Stated preferences for dog characteristics and sources of acquisition. Animals (Basel).

[16] Holland K.E (2019). Acquiring a pet dog:A review of factors affecting the decision-making of prospective dog owners. Animals (Basel).

[17] Amissah-Reynolds P.K, Monney I, Adowah L.M, Agyemang S.O (2016). Prevalence of helminths in dogs and owners'awareness of zoonotic diseases in Mampong, Ashanti, Ghana. J. Parasitol. Res.

[18] Ojo G.A, Adekeye T.A, Awobode H.O (2019). Prevalence of single and mixed parasitic infections of dogs in Egbeda communities, Ibadan, Oyo State, Nigeria. Sokoto J. Vet. Sci.

[19] Traversa D, di Regalbono A.F, Di Cesare A, La Torre F, Drake J, Pietrobelli M (2014). Environmental contamination by canine geohelminths. Parasit. Vectors.

[20] Dantas-Torres F, Ketzis J, Mihalca A. D, Baneth G, Otranto D, Perez Tort G, Watanabe M, Khanh Linh B, Inpankaew T, Jimenez Castro P.D, Borrás P, Arumugam S, Penzhorn B.L, Ybañez A.P, Irwin P, Traub R.J (2020). TroCCAP recommendations for the diagnosis, prevention and treatment of parasitic infections in dogs and cats in the tropics. Vet. Parasitol.

[21] Nguyen T, Clark N, Jones M.K, Herndon A, Mallyon J, Magalhaes R.J.S, Abdullah S (2021). Perceptions of dog owners towards canine gastrointestinal parasitism and associated human health risk in Southeast Queensland. One Health.

[22] Amaro A.A, Greer T, Wilson D, Smrdelj M (2022). Giant red kidney worm (*Dioctophyma renale*) infection in puppies less than four months of age from Northern Canada. J. Parasitol.

[23] Sprent J.F.A (1961). Post-parturient infection of the bitch with *Toxocara canis*. J. Parasitol.

[24] Ribeiro V.M, Júnior D.M.G, Ottino J, Valle G.R, de Miranda Estevam L.G.T, de Carvalho O.V, Paz G.F (2022). Report of the presence of *Leishmania infantum* in the milk of a naturally infected female dog in Brazil. Vet. Parasitol. Reg. Stud. Rep.

[25] Weese J.S, Fulford M.B (2011). Companion Animal Zoonoses.

[26] Schär F, Inpankaew T, Traub R.J, Khieu V, Dalsgaard A, Chimnoi W, Odermatt P (2014). The prevalence and diversity of intestinal parasitic infections in humans and domestic animals in a rural Cambodian village. Parasitol. Int.

[27] Kohansal M.H, Fazaeli A, Nourian A, Haniloo A, Kamali K (2017). Dogs'gastrointestinal parasites and their association with public health in Iran. J. Vet. Res.

[28] Contreras-Flores A.A, Romero-Castanon S, Rocha-Rocha V.M (2021). Gastrointestinal parasites in dog feces in Puebla City, Mexico. J. Adv. Parasitol.

[29] Traversa D (2012). Pet roundworms and hookworms:A continuing need for global worming. Parasit. Vectors.

[30] Palmer C.S, Robertson I.D, Traub R.J, Rees R, Thompson R.A (2010). Intestinal parasites of dogs and cats in Australia:The veterinarian's perspective and pet owner awareness. Vet. J.

